# The Cardiovascular and Metabolic Effects of Chronic Hypoxia in Animal Models: A Mini-Review

**DOI:** 10.3389/fphys.2022.873522

**Published:** 2022-03-31

**Authors:** Laura A. Barnes, Omar A. Mesarwi, Ana Sanchez-Azofra

**Affiliations:** ^1^ Division of Pulmonary, Critical Care, and Sleep Medicine and Physiology, Department of Medicine, University of California, San Diego, San Diego, CA, United States; ^2^ Servicio de Neumología, Hospital Universitario de la Princesa, Universidad Autónoma de Madrid, Madrid, Spain

**Keywords:** animal modeling, chronic hypoxia, cardiovascular disease, high altitude, sleep, metabolism

## Abstract

Animal models are useful to understand the myriad physiological effects of hypoxia. Such models attempt to recapitulate the hypoxemia of human disease in various ways. In this mini-review, we consider the various animal models which have been deployed to understand the effects of chronic hypoxia on pulmonary and systemic blood pressure, glucose and lipid metabolism, atherosclerosis, and stroke. Chronic sustained hypoxia (CSH)—a model of chronic lung or heart diseases in which hypoxemia may be longstanding and persistent, or of high altitude, in which effective atmospheric oxygen concentration is low—reliably induces pulmonary hypertension in rodents, and appears to have protective effects on glucose metabolism. Chronic intermittent hypoxia (CIH) has long been used as a model of obstructive sleep apnea (OSA), in which recurrent airway occlusion results in intermittent reductions in oxyhemoglobin saturations throughout the night. CIH was first shown to increase systemic blood pressure, but has also been associated with other maladaptive physiological changes, including glucose dysregulation, atherosclerosis, progression of nonalcoholic fatty liver disease, and endothelial dysfunction. However, models of CIH have generally been implemented so as to mimic severe human OSA, with comparatively less focus on milder hypoxic regimens. Here we discuss CSH and CIH conceptually, the effects of these stimuli, and limitations of the available data.

## Introduction

Animal models have been useful for demonstrating various physiological effects of hypoxia, thus providing deeper understanding of the impact of hypoxemia in human disease. Chronic sustained hypoxia (CSH) and chronic intermittent hypoxia (CIH) are each associated with cardiovascular and metabolic changes, which can be adaptive or maladaptive. In this mini-review we consider the outcomes associated with both CSH and CIH as they pertain to cardio-metabolic disease. Specifically, we will address cardiovascular and metabolic outcomes of CSH and CIH models in animals which aim to mimic human disease states. We will not focus on models of acute sustained or intermittent hypoxia (lasting minutes to hours), which may have variable consequences. Moreover, in this mini-review, we consider only CSH and CIH models which might resemble chronic hypoxic conditions in humans. Intriguing reports of the effects of intermittent hypoxia on neuroplasticity with low-frequency hypoxic episodes lasting several minutes (Gonzalez-Rothi et al., 2015; Navarrete-Opazo et al., 2015) are subjects of other expert reviews (Randelman et al., 2021). Finally, we note that our intention is to cover significant breadth of understanding of the topic of cardio-metabolic consequences of chronic hypoxia in animal models, sacrificing some depth of specific models and outcomes. We invite the reader to explore specific citations for important study details.

## Hypoxia as a Model of Human Disease

Both CSH and CIH in animal models have been used to simulate various disease states. CSH has been applied to rodents at varying fraction of inspired oxygen (FiO_2_), generally ranging from 0.10 to 0.15 (Hislop and Reid, 1976; Cowburn et al., 2017; Ioja et al., 2018; Prieto-Lloret et al., 2021), either been normobaric or hypobaric relative to sea level. Though resulting peripheral saturations are not always considered, the severity of CSH is a critical variable: For instance, CSH of FiO_2_ 0.10 as a model of high altitude exposure might recapitulate the effective oxygen content at an altitude of 5800 m (Mt. Kilimanjaro), whereas an FiO_2_ of 0.15 might be representative of a lower altitude (2400 m, Aspen, CO). In considering analogues of human disease, an FiO_2_ of 0.10 would be expected to model only very hypoxemic diseases like cyanotic heart disease, whereas an FiO_2_ of 0.15 might model chronic obstructive pulmonary disease (COPD), or other chronic lung diseases which are far more common.

Similarly, CIH has been applied to animal models in a variety of ways, although most studies roughly reproduce conditions used by Fletcher *et al.*, who first studied CIH in rodents as a model of OSA (Fletcher et al., 1992b). In CIH, multiple variables of desaturation and resaturation are important to define. In Fletcher’s experiments, rats were exposed to rapid reductions of FiO_2_ from 0.21 to 0.05 over 12 s, then quickly returned to 0.21. This process was repeated every 90 s (corresponding to an oxyhemoglobin desaturation index [ODI] of 40 events/h), for 7 h per day, for up to 5 weeks. Each of these variables—rate of deoxygenation, depth of deoxygenation, rate of reoxygenation, ODI, duration of daily exposure, and overall experiment duration—may be manipulated in different animal experiments (Farré et al., 2018). At least one study has demonstrated tissue-specific effects of various hypoxic profiles of CIH in rodents (Reinke et al., 2011). Thus, there are several considerations when designing animal experiments seeking to elucidate the physiological effects of either CSH or CIH.

## Cardio-Metabolic Effects of Chronic Sustained Hypoxia

### CSH and Pulmonary Hypertension

In humans and in animal models, acute alveolar hypoxia has been shown to cause pulmonary vasoconstriction, leading to acute pulmonary hypertension (PH) (Fishman, 1976; Wagenvoort, 1977; Rabinovitch et al., 1979; Perkin and Anas, 1984; Voelkel, 1986). Both hypoxic pulmonary vasoconstriction and PH may revert after cessation of hypoxic exposure. By contrast, exposure to CSH results in chronic PH which may be irreversible (Meyrick and Reid, 1978; Stenmark et al., 2009). Vascular remodeling due to CSH consists of muscularization of the small arteries of the alveolar wall and proliferation of cells expressing α-smooth muscle actin, followed by thickening of the precapillary pulmonary arteries, inflammation, and fibrosis of the large proximal pulmonary arteries (Stenmark et al., 2009). CSH causes PH so reliably in rodents that it has been widely adopted as a model for studying mechanisms and downstream effects of PH. However, the response to CSH is variable between species (Stenmark et al., 2009). Although CSH leads to PH both in mice and rats, the degree of vascular remodeling is typically less in mice (Hislop and Reid, 1976; Frank et al., 2008; Cahill et al., 2012).

### Sustained Hypoxia and Systemic Blood Pressure

While CSH causes PH in rodent models, the effect of CSH on systemic blood pressure is less clear. Acute ascent to high altitude, an inherently hypoxic environment, can reversibly increase systemic blood pressure (Bender et al., 1988; Wolfel et al., 1991, Wolfel et al., 1994). Epidemiological studies have shown that humans living at high altitude have *lower* systemic blood pressure than those living at sea level (Rotta, 1947; Ruiz and Peñaloza, 1977), highlighting the difference between acute exposure and those acclimatized to such an environment. In rodents exposed to normobaric or hypobaric CSH, results have been mixed. Vilar *et al.* demonstrated a reduction in blood pressure in spontaneously hypertensive rats after exposure to normobaric CSH (FiO_2_ of 0.10 for 8 weeks) (Vilar et al., 2008), induction of pro-angiogenic pathways; and they showed that neutralizing antibodies targeting vascular endothelial growth factor-A (VEGF-A) both abrogated the effects of hypoxia on angiogenesis, and increased blood pressure. Other studies also showed that CSH decreased systemic blood pressure in young spontaneously hypertensive rats (Henley and Tucker, 1987), and that hypoxia mitigated blood pressure elevation in the renal hypertensive rat (Fregly, 1963, Fregly, 1970). However, one study demonstrated that CSH (FiO_2_ of 0.10) did change blood pressure in male rats at durations of anywhere from 1 to 30 days, despite an increase in carotid body catecholaminergic signaling (Hui et al., 2003). Our group has also not observed changes in systemic blood pressure in young mice exposed to 40 days of CSH of similar severity (Zhen et al., 2021). Vaziri *et al.* demonstrated *increased* blood pressure in rats exposed to hypobaric CSH (effective FiO_2_ of 0.10–0.11) that persisted even after the restoration of normoxia (Vaziri and Wang, 1996). Thus, the effects of CSH on systemic blood pressure are complex, and perhaps dependent on the specific conditions and animals.

### Effects of CSH on Atherosclerosis and Stroke

Atherosclerosis is the major underlying etiology of cardiovascular disease, which is the leading cause of death worldwide (Mendis et al., 2011). Evidence for the contribution of hypoxia to the progression of atherosclerosis is largely circumstantial. Hypoxia inducible factor-1α (HIF-1α), a subunit of HIF-1, the major regulator of the cellular response to hypoxia, is normally quickly hydroxylated and degraded in normoxia. In hypoxia, however, HIF-1α is stabilized and can dimerize with HIF-1β, allowing binding to hypoxia responsive elements in the promoter regions of target genes of interest (Iyer et al., 1998). HIF-1α is stabilized in macrophages and smooth muscle cells near the necrotic core of atherosclerotic vascular lesions in humans and in animal models (Sluimer et al., 2008; Lim et al., 2013; Ferns and Heikal, 2017), and HIF-1 has been implicated in atherosclerosis progression (Kasivisvanathan et al., 2011). Moreover, hyperbaric oxygen (FiO_2_ 1.0, 2.4–2.5 atm) improves atherosclerosis in both rabbits and mice (Kudchodkar et al., 2000, Kudchodkar et al., 2007, Kudchodkar et al., 2008). It is therefore conceivable that hypoxia could contribute to the development of atherosclerosis, but to our knowledge, CSH has never been shown to directly impact atherosclerosis in animal models.

Atherosclerosis, among other factors, may lead to acute ischemic stroke, which causes over 130,000 deaths in the United States yearly. Patients with pre-existing atherosclerotic lesions who then become hypoxemic (e.g., respiratory failure in the ICU setting) may develop sufficient brain ischemia to manifest as a stroke. However, recent data suggest that acute hypoxic exposure in animal models of ischemic stroke may be protective. Mice with stroke induced by middle cerebral artery occlusion and then exposed to variably severe hypoxia (FiO_2_ of 0.07–0.12) for two to 8 weeks (Zhang et al., 2020) showed improved collateral blood flow in a “dose-dependent” manner, with more severe and longer duration of hypoxia generating more robust collateral circulation. These effects were durable even after cessation of hypoxia. These data suggest that while some effects of CSH may be maladaptive, some might be beneficial, and that adaptive responses to hypoxia may present in unique ways.

### Metabolic Effects of CSH

Despite our ability to implement CSH as a stimulus with relative ease in animal studies, the metabolic effects of CSH are less well explored than the cardiovascular effects. Gamboa *et al.* were the first to recognize the potentially beneficial effects of CSH on glucose metabolism (Gamboa et al., 2011), finding that CSH with an FiO_2_ of 0.10 reduced plasma fasting glucose and insulin, increased insulin sensitivity, and improved insulin-dependent glucose uptake by skeletal muscle. Since that time, similar findings have been replicated by us (Zhen et al., 2021) and others (Lee et al., 2013; Ioja et al., 2018), with additional data demonstrating hypoxia-dependent effects on the liver transcriptome (Zhen et al., 2021) and changes in liver and skeletal muscle mitochondrial function (Ioja et al., 2018). Lipid metabolism also appears to be altered in CSH, with elevated serum triglyceride and low-density lipoprotein levels resulting from CSH with an FiO_2_ of 0.10 (Zhen et al., 2021). We and others have noted that CSH causes weight loss in rodents. In our studies, however, we found a complex interaction between hypoxia and weight, and that beneficial metabolic effects of CSH cannot solely be explained by weight reduction (Zhen et al., 2021).

## Cardio-Metabolic Effects of Chronic Intermittent Hypoxia

CIH has been used to model OSA, the most common respiratory disease in the world (Benjafield et al., 2019). Epidemiologic associations have been made between OSA and a wide variety of adverse health outcomes, including cardiovascular disease, diabetes, cognitive and mood disorders, and others. However, OSA has several significant manifestations aside from intermittent hypoxemia, including hypercapnia, intrathoracic pressure swings, and fragmented sleep. CIH models attempt to understand the mechanisms by which the hypoxemia of OSA may uniquely contribute to these outcomes of interest.

### CIH and Pulmonary Hypertension

OSA in humans is associated with PH, although the effect is typically mild (Sajkov and McEvoy, 2009) and the impact of OSA on PH independent of other comorbidities has been debated (Chaouat et al., 1996; Sajkov et al., 1999). In OSA, the duration of hypoxemia resulting from respiratory events (apneas or hypopneas), rather than the frequency of respiratory events as gauged by the apnea-hypopnea index *per se*, is linked with more severe pulmonary hypertension (Samhouri et al., 2020). In early animal models involving dogs, repetitive airway occlusion was induced by tracheal obstruction of variable duration. These studies showed that pulmonary arterial (PA) pressure increased as a function of the severity of desaturation (Iwase et al., 1992). Further, the authors showed that airway occlusion in animals allowed to breathe 100% oxygen (which prevented significant desaturations), did not increase PA pressure. Likewise, when another set of dogs were allowed to breathe hypoxic gas without airway occlusion, PA pressures increased. These observations suggested that hypoxemia is likely the most critical of the several physiological manifestations of OSA to cause PH. There are several studies examining the impact of CIH on pulmonary hypertension in rodent models (Fagan, 2001; McGuire and Bradford, 2001; Campen et al., 2005; Nisbet et al., 2009; Nara et al., 2015; Snow et al., 2020; Zhen et al., 2021). Some of these studies appear to show increases in right ventricular systolic pressure, right ventricular mass, and/or pulmonary vascular remodeling in response to CIH, although we did not observe these effects in young C57BL/6J mice (Zhen et al., 2021). CIH also does not increase right ventricular pressures to the same degree as CSH (Fagan, 2001; Zhen et al., 2021). Any putative effect of CIH to worsen pulmonary hypertension may be due to changes in nitric oxide bioavailability (Nisbet et al., 2009) and/or increases in endothelin-1 expression and endothelial damage (Wang et al., 2013), leading to pulmonary vasoconstriction.

### CIH and Systemic Blood Pressure

As mentioned above, the first demonstrated physiological effects of CIH were to increase systemic blood pressure in rats (Fletcher et al., 1992b). Since that time, this finding has been demonstrated by others (Fletcher, 2001; Prabhakar and Kumar, 2010). The major mechanism by which CIH is thought to induce hypertension is by activation of the sympathetic nervous system. Fletcher *et al.* showed that surgical denervation of peripheral chemoreceptors in the carotid body prevented CIH-induced elevations in arterial blood pressure in rats (Fletcher et al., 1992a). CIH also impairs endothelium-dependent vasodilation of skeletal muscle resistance arteries (Tahawi et al., 2001; Phillips et al., 2004) and causes vascular remodeling (Fletcher et al., 1992b). CIH increases the responsiveness of the carotid body to hypoxia, causing upregulation of pro-inflammatory cytokines, and activation of the sympathetic nervous system (Lesske et al., 1997; Braga et al., 2006; Dick et al., 2007; Lam et al., 2012, Lam et al., 2014; Zoccal et al., 2019). HIF-1 has also been implicated in the development of hypertension in animal models, via downstream effects on nicotinamide adenine dinucleotide phosphate (NADPH) oxidase (Yuan et al., 2011; Schulz et al., 2014; Semenza and Prabhakar, 2015). The CIH-mediated increase in blood pressure persists even after cessation of CIH, and CIH may increase blood pressure in male rats in as little as 2 days of exposure (Hinojosa-Laborde and Mifflin, 2005). Our group has noted that there may be acclimatization to CIH causing normalization of blood pressure over prolonged periods (4–5 weeks) (Zhen et al., 2021), but more work is needed to define this effect further.

### Effects of CIH on Atherosclerosis and Stroke

While exposing wild-type mice to CIH may induce vascular inflammation and remodeling (Gileles-Hillel et al., 2014), it does not appear to result in overt atherosclerosis (Savransky et al., 2007b; Drager et al., 2013), even after prolonged exposure (e.g., 20 weeks) (Song et al., 2012). However, atherosclerosis is observed in wild-type mice fed a high cholesterol diet in conjunction with exposure to CIH (Savransky et al., 2007b). Additionally, in studies using ApoE-deficient mice, which are more susceptible to atherogenesis, CIH exposure induces atherosclerosis (Jun et al., 2010; Arnaud et al., 2011; Fang et al., 2012; Gautier-Veyret et al., 2013). The major mechanism for the development of atherosclerosis in CIH appears to be the excess expression of pro-inflammatory pathways. Nuclear factor kappa B (NF-κB) is important for the development of atherosclerosis in rodents exposed to CIH (Fang et al., 2012; Song et al., 2018), and HIF-1 also may play a role in the development of CIH-induced atherosclerosis (Drager et al., 2013; Zhou et al., 2014).

Compared to the outcomes mentioned above, few animal studies have examined the relationship between CIH and stroke, even though human epidemiological studies have strongly linked OSA to stroke risk (Dyken et al., 1996; Yaggi et al., 2005; Das and Khan, 2012). CIH increases the brain’s susceptibility to hypoxia by altering cerebral blood flow (Phillips et al., 2004; Capone et al., 2012). Mechanisms for this include increased endotheliln-1 and increased oxidative stress via NADPH oxidase (Capone et al., 2012). Canzani *et al.* demonstrated that intermittent airway obstruction increased reperfusion injury in a mouse model of ischemia-reperfusion injury (Cananzi et al., 2020). Another intriguing study showed that CIH with a nadir FiO_2_ of 0.10 may be neuroprotective, whereas a nadir FiO_2_ of 0.06 may exacerbate neurological damage (Jackman et al., 2014), suggesting that the specific model of CIH, mimicking a specific severity of OSA, is fundamentally important.

### Metabolic Effects of CIH

CIH also reliably impacts glucose and lipid metabolism. CIH induces insulin resistance and glucose intolerance in obese mice, whether due to diet or genetic modification (leptin-deficient Ob^−^/Ob^−^ mice) (Polotsky et al., 2003; Drager et al., 2011). We and others have noted similar effects of CIH in lean mice (Iiyori et al., 2007; Zhen et al., 2021). Although some groups have noted either sex-specific effects of CIH on glucose metabolism (Marcouiller et al., 2021), or improvement in glucose tolerance with CIH (Polotsky et al., 2003; Carreras et al., 2012; Thomas et al., 2017), this is usually accompanied by an increase in whole-body insulin resistance, suggesting the complexity of the response to CIH on glycemia, which may at best be mixed, and in some scenarios deleterious. Additionally, CIH worsens nonalcoholic fatty liver disease and other types of liver injury in mice with diet-induced obesity (Savransky et al., 2007a, Savransky et al., 2007c; Mesarwi et al., 2021), and alters lipid metabolism (Drager et al., 2012; Jun et al., 2012; Yao et al., 2013; Zhen et al., 2021). It is important to note that the CIH model used in these studies is frequently designed to simulate severe OSA—that is, with severe reductions in nadir FiO_2_ (0.04–0.07), and a high ODI. The effects of less severe CIH on glucose and lipid metabolism are not well described.

## Future Directions

Though much has been accomplished regarding our understanding of the diverse cardio-metabolic consequences of CSH and CIH, there are clearly areas which merit further investigation. First, there are gaps in our understanding of the physiological effects of milder CIH and CSH. CSH has been investigated mostly with an FiO_2_ of 0.10, which likely represents a level of hypoxemia more severe than commonly observed in chronic heart/lung diseases in humans. It has been suggested that one might expect adaptive, rather than maladaptive, physiological responses to milder CIH (Navarrete-Opazo and Mitchell, 2014). Second, some of the outcomes presented in this mini-review have only a minimal amount of accompanying mechanistic data; there is undoubtedly room to devote more complete exploration of these concepts. Third, in particular when considering effects of CSH, one must consider whether normobaric hypoxia differs from hypobaric hypoxia, which has relevance for studies involving physiological outcomes of CSH models intended to mimic exposure to high altitude. Although there has been debate about this topic for years (Millet et al., 2012; Mounier and Brugniaux, 2012), animal studies examining the effects of CSH are typically not performed in both conditions, creating uncertainty about the impact of atmospheric pressure on the outcome being measured. Indeed, the uncertainty on this point extends to human-based research as well (Coppel et al., 2015). Finally, in our group, we have noted unique cardio-metabolic consequences of combined sustained and intermittent hypoxia, or “overlap hypoxia”, which may be used to model the COPD/OSA overlap syndrome, or periodic breathing at high altitude (Zhen et al., 2021). A systematic approach to understanding the hypoxemia of this unique condition is needed.

## Conclusion

Both CSH and CIH are associated with unique, and sometimes maladaptive, physiological responses, though there are considerable differences between these types of hypoxic exposures. CSH and CIH are intended to mimic hypoxemia in human disease states, but the heterogeneity of hypoxemia severity in cardiovascular and pulmonary disease mandates that attention be given to novel and more nuanced models. Future work can be directed toward these goals, as well as toward better understanding of the mechanisms by which hypoxia alters cardio-metabolic physiology in animals.
